# Identification of Sweetness Preference-Related Single-Nucleotide Polymorphisms for Polygenic Risk Scores Associated with Obesity

**DOI:** 10.3390/nu16172972

**Published:** 2024-09-03

**Authors:** Ji Hyun Bae, Hyunju Kang

**Affiliations:** Department of Food Science and Nutrition, Keimyung University, Daegu 42601, Republic of Korea; jhb@kmu.ac.kr

**Keywords:** genome-wide association study, polygenic risk scores, sweetness preference, obesity, time-varying Cox regression

## Abstract

Our study aimed to identify sweetness preference-associated single-nucleotide polymorphisms (SNPs), characterize the related genetic loci, and develop SNP-based polygenic risk scores (PRS) to analyze their associations with obesity. For genotyping, we utilized a pooled genome-wide association study (GWAS) dataset of 18,499 females and 10,878 males. We conducted genome-wide association analyses, functional annotation, and employed the weighted method to calculate the levels of PRS from 677 sweetness preference-related SNPs. We used Cox proportional hazards modeling with time-varying covariates to estimate age-adjusted and multivariable hazard ratios (HRs) and 95% confidence intervals (CIs) for obesity incidence. We also tested the correlation between PRS and environmental factors, including smoking and dietary components, on obesity. Our results showed that in males, the TT genotype of rs4861982 significantly increased obesity risk compared to the GG genotype in the Health Professionals Follow-up Study (HPFS) cohort (HR = 1.565; 95% CI, 1.122–2.184; *p* = 0.008) and in the pooled analysis (HR = 1.259; 95% CI, 1.030–1.540; *p* = 0.025). Protein tyrosine phosphatase receptor type O (PTPRO) was identified as strongly associated with sweetness preference, indicating a positive correlation between sweetness preference and obesity risk. Moreover, each 10 pack-year increment in smoking was significantly associated with an increased risk of obesity in the HPFS cohort (HR = 1.024; 95% CI, 1.000–1.048) in males but not in females. In conclusion, significant associations between rs4861982, sweetness preference, and obesity were identified, particularly among males, where environmental factors like smoking are also correlated with obesity risk.

## 1. Introduction

The prevalence of obesity in the United States increased to 42.4% in 2018, and obesity-related metabolic syndromes, including type 2 diabetes, cardiovascular diseases, and cancer, are among the leading causes of premature death in this country [1]. Genome-wide association studies (GWAS) have revealed over 40 genetic variants associated with obesity since 2006, and most cases of obesity have multifactorial causes stemming from complex interactions between the associated genes and environmental factors, including dietary components [2]. Because the sense of taste plays a central role in the development of obesity (as it contributes to food selection) and, consequently, body weight, studies have been conducted to identify genetic predispositions to obesity in terms of polymorphisms that influence taste receptors. In humans, taste sensations arise when molecules from foods bind to taste receptors in the taste buds and gut cells [3]. The stimulation of taste receptors can also occur in extra-oral tissues [4], focusing attention on the relationship between taste receptors and obesity. However, not everyone experiences the same taste sensations due, in part, to individual genetic differences that influence food preferences and consumption.

Accumulating evidence has shown that susceptibility to obesity is governed by various genetic variants of taste receptors and their interactions with environmental factors [5,6], highlighting the importance of taste genetics. Taste-related single-nucleotide polymorphisms (SNPs) that are associated with taste perceptions in individuals with metabolic syndrome have been identified, revealing a strong inverse association between overall taste perceptions and body mass index (BMI) [7]. Although our understanding of obesity genetics has advanced significantly over the past few decades, the link between sweetness preference-associated receptors and obesity and the molecular mechanisms underlying the disease remain largely unknown. Additionally, although sex differences have been discovered in terms of caloric expenditure, body fat mass, and the onset of menopause [8], the understanding of how sex differences and SNPs in sweetness preference-associated receptors correlate with obesity is lacking. Given that obesity is a chronic disease due to an increase in body fat accumulation, high-sugar diets related to sweetness preference can lead to obesity. This is because sugar consumption increases body adiposity without causing a dramatic increase in body weight [9,10]. Therefore, our main goal was to perform a GWAS to identify sweetness preference-associated SNPs, characterize novel candidate genetic loci related to sweetness preference, and develop polygenic risk scores (PRS) based on sweetness preference-related SNPs to analyze their associations with obesity.

Our hypothesis was that sweetness preference-associated receptor polymorphisms are correlated with sex-dependent obesity and that the correlations between sweetness preference-associated receptor SNPs and environmental factors like smoking affect the pathogenesis of obesity. Thus, novel candidate loci associated with obesity and sweetness preference were identified and characterized within the American Nurses’ Health Study (NHS1) and Health Professionals Follow-up Study (HPFS) cohorts to verify the hypothesis. The results will have implications for diagnosing obesity, refining trial recruitment strategies, treating the disease, and tailoring personalized nutrition.

## 2. Materials and Methods

### 2.1. Study Populations

The study population included two large prospective cohorts (the NHS1 and HPFS cohorts). We followed up with the participants in both cohorts with biennial questionnaires regarding their medical histories and lifestyles, and semi-quantitative food-frequency questionnaires (FFQs) were completed every 4 years. The baseline year in both cohorts was 1986, when detailed information regarding the participants’ dietary habits and lifestyles was available. This study included 18,499 females and 10,878 males of European ancestry who had complete baseline information and available genotype data for GWAS [11,12,13]. All participants were free from diabetes and cancer at the baseline. We excluded both females and males with a BMI (in kg/m^2^) > 30 at baseline, as well as individuals with missing or implausible FFQ responses. Following exclusion based on these criteria, the final cohort used for data analysis included 12,098 females and 7555 males.

This study was approved by the institutional review board of Keimyung University (approval number 40525-202002-BR-087-01), and the protocol was reviewed and approved by the Brigham and Women’s Hospital and the Harvard T. H. Chan School of Public Health.

### 2.2. Assessment of Obesity and Covariates

Height and body weight data were self-reported in the questionnaires administered at enrollment and at each follow-up. The BMI was calculated as the kg/m^2^, and subjects with a BMI greater than 30 kg/m^2^ were determined to be obese. We converted the average time spent per week participating in physical activities (e.g., walking, running, and biking) to metabolic equivalent h (METs) per week [14]. Alcohol intake was assessed on the FFQ every 4 years, and trans fat and total energy intakes were updated from these questionnaires.

### 2.3. Dietary Assessment and Phenotype Definitions

The participants were asked about their frequencies of consuming specific foods and beverages during the past year, and the participants’ food-intake frequencies were quantified using nine categories: “never/almost never”, “one to three times a month”, “once a week”, “two to four times a week”, “five to six times a week”, “once a day”, “two to three times a day”, “four to five times a day”, and “more than six times a day” [15]. Sweetness preference was classified into two groups by calculating the cumulative average sum intake of sweet foods such as chocolate, cookies, brownies, donuts, cake, and jam. In this manner, two groups were created: “low” sweetness preference (<5 servings per month) and “high” sweetness preference (>50 servings per month) to determine the sweet-taste preference phenotype [16]. The grouping rationale was based on previous findings showing that the recalled sweet taste intensity was associated with self-reported liking and habitual intake of commonly consumed sweet foods [17].

### 2.4. Genotyping and Calculating PRS

Genotyping and merging were performed with the pooled GWAS dataset, which was generated using five different platforms (Illumina HumanHap array, Illumina OmniExpress array, Humancore array, Oncoarray, and Affymetrix 6.0 array), as described in detail by Lindström et al. [18]. Samples with a missing call rate >5% (with any platform) during the merging process were excluded from further analysis. SNPs with a minor allele frequency <1% or an imputation quality (r^2^) <0.5 were also excluded [19]. Missing genotypes were imputed using the Haplotype Reference Consortium as the reference panel [20].

We used a weighted method to calculate the PRS based on 677 SNPs (*p* < 1.0 × 10^−3^). Each SNP was weighted by its relative effect size (β coefficient) on the sweetness preference. We calculated the PRS using the equation weighted PRS = (β1 × SNP1 + β2 × SNP2 + … + β677 × SNP677) × (677/sum of the β coefficients of all SNPs), where SNPi is the risk allele number of each SNP, to ensure that it accurately reflects the risk [21].

### 2.5. Functional Annotation and Gene Mapping

Functional annotation was performed using Functional Mapping and Annotation of Genome-Wide Association Studies (FUMA GWAS, an online platform for the functional mapping of genetic variants) [22]. Additionally, Combined Annotation Dependent Depletion (CADD) [23] scores (with scores > 12.37 indicating the deleteriousness of an SNP) and RegulomeDB [24] scores (with a lower score indicating a higher probability of having a regulatory function) were annotated to SNPs. The nearest gene to each SNP was identified using HaploReg v4.1, a tool for exploring annotations of the noncoding genome in variants on haplotype blocks, using the RefSeq genes [25].

### 2.6. Statistical Analysis

Genome-wide association analyses were performed using PLINK [26,27] and a logistic-regression model for the two pooled GWAS datasets. A meta-analysis was conducted using METAL software [28]. We used Cox proportional hazards modeling with time-varying covariates to estimate age-adjusted and multivariable hazard ratios (HRs) and 95% confidence intervals (CIs) for incident obesity. Each participant’s person-time of follow-up was calculated based on the return date of the baseline questionnaire (i.e., 1986 for the NHS and HPFS) to the date of obesity diagnosis based on the BMI value (>30 kg/m^2^), death, or the end of follow-up, whichever occurred first [29]. Males and females who reported a BMI > 30 kg/m^2^, had cancer or diabetes, or who died were excluded from the subsequent follow-up. Among the covariates, the age, level of physical activity, trans fat level, and total energy intake were added to the model as continuous variables. The smoking status and alcohol consumption level were added as categorical variables. Interaction effects of the PRS and environmental factors on incident obesity were also tested by including the interaction terms in the regression models. Adjusted multivariable HRs for both cohorts were pooled using fixed-effect meta-analysis, using either SAS software (version 9.3; SAS Institute Inc., Cary, NC, USA) or R software (version 4.0.0; R Foundation for Statistical Computing, Vienna, Austria) [30].

## 3. Results

### 3.1. Sweetness Preference GWAS Analysis

**Figure 1:**
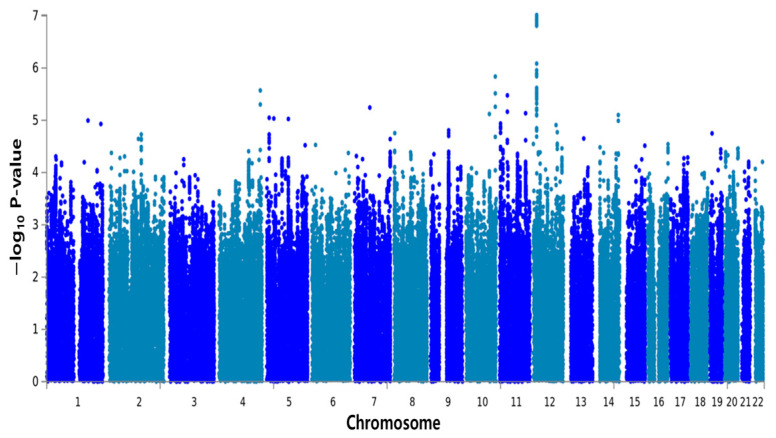
Manhattan plot displaying significant SNPs according to their −log_10_ *P*-values (shown on the y-axis). The SNPs are ordered by their chromosomal position along the x-axis. Each dot on the Manhattan plot signifies an SNP, and the SNP with the strongest association (rs1457538), i.e., with the smallest P-value (*p* = 9.68 × 10^−^^8^), was on chromosome 12.

In the discovery set of the GWAS, we further selected the 11 lead SNPs that were most significantly associated with sweetness preference (*p* < 1.0 × 10^−5^) (Table 1). The most significant SNP identified in the combined set was on chromosome 12 (rs1457538, *p* = 9.68 × 10^−8^), with the nearest gene being protein tyrosine phosphatase receptor type O (PTPRO). One SNP was identified on chromosome 4 (rs4861982, *p* = 2.68 × 10^−6^), with the nearest gene being long intergenic non-protein-coding RNA 290 (LINC00290). Two SNPs were also identified on chromosome 11: rs11606257 (*p* = 3.34 × 10^−6^, nearest gene: apoptotic peptidase activating factor 1 interacting protein [APIP]), which had the highest CADD score (13.03) among all SNPs, and rs220850 (*p* = 7.33 × 10^−6^, nearest gene: cell adhesion molecule 1 [CADM1]). Figure 1 shows a Manhattan plot for the genome-wide meta-analysis of SNPs related to sweetness preference in all study participants. The most strongly associated SNP, rs1457538, with the smallest P-value (*p* = 9.68 × 10^−8^) was on chromosome 12.

### 3.2. Characteristics According to PRS between Males and Females

Baseline age-adjusted descriptive statistics were determined according to the highest, intermediate, and lowest thirds of the PRS in the NHS1 and HPFS cohorts (Table 2). Females were more likely to be current smokers at baseline. Males generally consumed higher levels of alcohol, had higher total energy intake, a higher level of trans fat intake, and greater total fiber intake, whereas females consumed more coffee, fruits, and sweetened beverages at baseline. Males and females had similar healthy eating indexes and sleep times. However, the males had higher glycemic load levels than the females and consumed more ice cream, chocolate, and cake at baseline. The thirds of the PRS values ranged from 631 (low) to 665 (high), and the participants with higher PRS had higher intakes of chocolate, ice cream, and cake.

### 3.3. Genotype of SNP rs4861982 and Obesity

**Figure 2:**
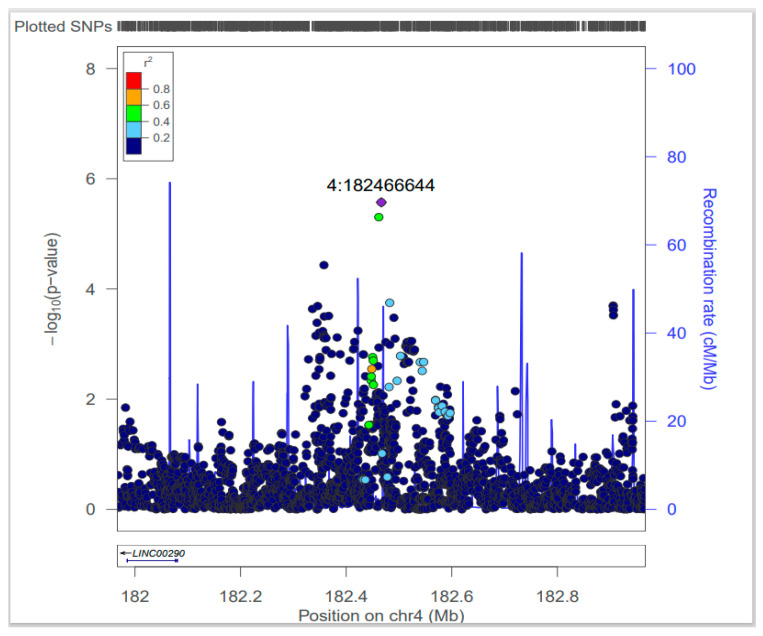
Regional association plot for the reference SNP, rs4861982. SNPs are plotted by displaying the negative logarithm of the associated P-value as a function of the genomic position. The SNP rs4861982 is located on chromosome 4 at nucleotide position 182466644 (based on GRCh37); it was identified as the most strongly associated SNP on chromosome 4 (rs4861982, purple diamond).

Among the SNPs associated with sweetness preference, rs4861982 was associated with obesity in the NHS1 and HPFS cohorts (Table 3). In males, the TT genotype of rs4861982 significantly increased the risk of obesity as compared to the GG genotype in HPFS cohort in Model 1 (HR = 1.596; 95% CI, 1.145–2.225; *p* = 0.006) and Model 2 (HR = 1.565; 95% CI, 1.122–2.184; *p* = 0.008). Similar results were obtained in the pooled analysis in Model 1 (HR = 1.262; 95% CI, 1.032–1.543; *p* = 0.023) and Model 2 (HR = 1.259; 95% CI, 1.030–1.540; *p* = 0.025). In females, however, the TT genotype was not significantly associated with obesity. The association of the TG genotype of rs4861982 with obesity was negligible in both males and females, as well as in the pooled analysis. A regional association plot for the reference SNP rs4861982 is shown in Figure 2. SNPs are plotted with the negative logarithm of the associated P-value as a function of the genomic position ranging from chr4:181966644 to chr4:182966644 (Genome Reference Consortium Human Build 37, GRCh37). The most strongly associated SNP was found on chromosome 4 at nucleotide position 182466644 (rs4861982, purple diamond) [31].

### 3.4. Correlation Effects of PRS and Environmental Factors on Obesity

**Figure 3:**
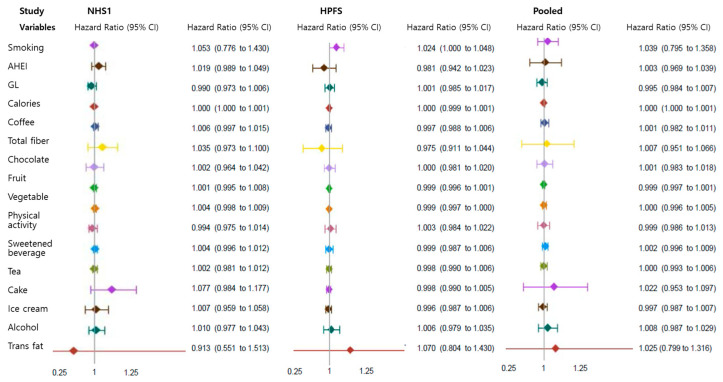
Correlation of PRS with environmental factors (such as lifestyle and dietary components) in the HR of obesity. Different colors represent distinct variables, as indicated in the figure. The forest plots show HRs and 95% CIs for interactions between the PRS (per 10 risk alleles) and changes in environmental factors (10-increment servings per month). The results are adjusted for the same set of variables shown in Table 3. The results for two cohorts are pooled by means of inverse variance-weighted, fixed-effects meta-analysis. All P-values for heterogeneity are >0.05.

Different socioeconomic or cultural factors [32] and family environments can increase the prevalence of obesity through food supply, caloric intake, and physical activity [33]. Therefore, we further investigated the ability of genetic analysis, in the form of a PRS, to identify individuals who are at high risk of obesity. Correlation analyses between the PRS and environmental factors, such as smoking and dietary components, are summarized in Figure 3. Including the Alternate Healthy Eating Index (AHEI), glycemic load (GL), other dietary factors, and physical activity in our models did not show significant PRS interactions with the risk of obesity. No consistent correlation effects were found between any type of dietary intake and the PRS regarding obesity risk. However, each 10 pack-year increment of smoking, in the interaction with PRS per additional 10 risk alleles, was associated with an increased risk of obesity in the HPFS cohort (HR = 1.024, 95% CI, 1.000–1.048), as shown in Figure 3. These findings indicate that smoking is significantly associated with a correlation between the PRS and obesity in males.

## 4. Discussion

CTNND2, the gene identified here, is expressed within proliferating neuronal progenitor cells of the neuroepithelium and in the dendritic compartment of postmitotic neurons [34]. Genetic variation in CTNND2 has been reported to be involved in neuroplastic processes in the olfactory pathways of rats [35] and is associated with human neurodevelopmental phenotypes, such as autism [34] and intellectual disability [36]. CTNND2 is identified as an adhesion-related molecule in human periodontal ligament cells [37] and is located at 5p15.2, predominantly expressed in the brain with distinct regional expression patterns [38]. Despite several genome-wide studies implicating polymorphisms within CTNND2 [39], a definitive role of CTNND2 in the pathogenesis of obesity has not yet been determined.

Several genes have been reported to be related to obesity in previous studies. Among these genes, WBP1L was found to be related to BMI in a meta-analysis of the epigenome-wide association study in REGICOR (REgistre GIroní del COR) [40]. The expression of the cell-proliferation marker MKI67 was also significantly increased in the endometrial polyps of postmenopausal females with obesity, suggesting that the BMI influences the proliferation marker [41]. The SNP most strongly associated with sweetness preference, with the smallest *P*-value (*p* = 9.68 × 10^−8^), is rs1457538 on chromosome 12 (Table 1). The nearest gene to rs1457538 is PTPRO, which is a member of the R3 subfamily of receptor-like protein tyrosine phosphatases and is associated with adipose tissue. The PTPRO gene is upregulated in the adipose tissues of obese individuals [42]. The roles of PTPRO have been reported to involve the control of glucose and lipid metabolism, obesity-induced systemic inflammation [43], and the inactivation of the insulin receptor [44]. Although the heterogeneity (I²) for rs1457538 is relatively high, the expression of PTPRO may contribute to the risk of obesity, implying a positive correlation between sweetness preference and obesity risk.

One important finding of this study is that the closest gene to rs4861982 (*p* = 2.68 × 10^−6^; implicated in this study) is LINC00290, a long intergenic non-protein-coding RNA gene. Interestingly, LINC00290 interacts with sodium arsenite, a naturally occurring component of sediment and groundwater, which makes human exposure inevitable [45]. Both arsenic exposure and obesity are prevalent and widespread [46], and arsenic exposure can affect gene regulation at both the transcriptional-initiation and splicing levels [45].Coincidentally, our epidemiological study of rs4861982, which revealed its correlation with sodium arsenite, showed that it significantly increased the risk of obesity in males but not in females. Given that this cohort study was based on a subset of the NHS1 and HPFS with an age range of 50–67 years, it is plausible that the GWAS signal indicating that the TT genotype was significant only in males was partially influenced by the onset of menopause, postmenopausal hormonal changes between females and males, and socioeconomic status when comparing females with males [47,48].

Studies have shown that energy imbalance and metabolic disorders can lead to obesity by affecting other contributing or predisposing factors. The interplay between obesity and environmental factors, such as obesogenic infectious agents, toxic chemicals, genetic influences, epigenetic influences, the gut microbiome, and brown or beige fat, can make certain groups more susceptible to obesity [9]. Environmental factors like smoking can affect energy balance and metabolism, thereby increasing the risk of obesity. We found a significant and unique gene-environment interaction of the PRS with smoking in males, where males who smoked more had an approximately 2.4% increased risk of obesity. This result indicates that smoking masks the PRS effect, which typically results in weight loss, yet it contributes to a mere 2.4% increase in the obesity risk, whereas the TT genotype of rs4861982 contributes to a significantly higher risk of obesity (56.5%). Further, cross-sectional studies indicate that the mean BMI tended to be lower among smokers than among nonsmokers in many populations [49]. We found no significant correlation between the PRS and female smokers. This may be because the TT genotype was significant only in males, partially due to the onset of menopause and postmenopausal hormonal changes, as mentioned above [47,48]. An important aspect of this study was the discovery that genome-wide PRS, along with a single SNP, can quantify hereditary obesity and identify adults at risk for obesity based on sex. While many studies on the association of a single SNP or genotype with obesity, this study uniquely investigated the effect of PRS on obesity as well as the correlated effects of environmental factors.

A limitation of this study is that measurement errors of self-reported behaviors are inevitable. The inherent biases associated with self-reporting require educating participants on how to use the devices involved in data acquisition. Since one’s BMI can be estimated indirectly, individuals of different heights or body builds with different proportions of total body fat may exhibit similar BMI scores [50], which can provide limitations for this study. Additionally, the limitations that stem from cohort specificity, environmental factors, and gender disparity need to be addressed to overcome the heterogeneity of SNPs and their nearby genes related to obesity. This could be achieved by maximizing the sample size and refining samples by focusing on specific variables at onset and recurrence. The molecular mechanisms in which SNPs related to sweetness preference and related genes participate in the pathogenesis of obesity need to be further studied in the future.

## 5. Conclusions

This study verified that sweetness preference-related polymorphisms are associated with sex-dependent variation in obesity and that correlations between sweetness preference-related SNPs and environmental factors affect obesity. Specifically, significant associations between rs4861982 and both sweetness preference and obesity were identified. Compared to the GG or TG genotype, the TT genotype of rs4861982 significantly increased the risk of obesity among males. Among environmental factors, smoking was significantly associated with the correlations between the PRS and obesity in males but not in females.

## Figures and Tables

**Table 1:** Sweet taste preference GWAS analysis.

Lead SNPs	Adjacent Gene	CHR	BP	Minor Allele	Major Allele	MAF	OR (95% CI)	*P*-Value	Q	I^2^	CADD	RDB	eQTL (Tissue)
rs4861982	LINC00290	4	182466644	T	G	0.15	1.18 (1.10–1.26)	2.68 × 10^−6^	0.45	0	0.35	6	LINC00290 (Adrenal gland, Brain)
rs3891675	CTNND2	5	11537389	G	A	0.33	1.13 (1.07–1.19)	8.95 × 10^−6^	0.10	38.22	1.55	NA	
rs17512228	PDZD2	5	32071267	T	C	0.07	1.25 (1.13–1.37)	9.23 × 10^−6^	0.41	3.01	1.12	5	
rs2032890	ERAP1	5	96121152	C	A	0.30	0.88 (0.84–0.93)	9.43 × 10^−6^	0.79	0	7.63	4	CAST (Adipose_Visceral_Omentum, Brain) ERAP1 (Adipose_Subcutaneous)
rs11771792	AUTS2	7	68458785	C	T	0.17	1.17 (1.09–1.25)	5.72 × 10^−6^	0.65	0	1.81	5	
rs2778038	WBP1L	10	104515069	C	A	0.22	1.15 (1.08–1.22)	7.57 × 10^−6^	0.75	0	6.52	6	AS3MT (Adipose_Subcutaneous, Brain), WBP1L (Muscle_Skeletal, Brain) SFXN2 (Adipose_Visceral)
rs11596125	MKI67	10	130587803	A	G	0.24	1.15 (1.09–1.22)	1.45 × 10^−6^	0.25	20.74	8.36	5	
rs11606257	APIP	11	34933982	C	T	0.07	1.25 (1.14–1.38)	3.34 × 10^−6^	0.40	4.52	13.03	7	PDHX (Muscle_Skeletal)
rs220850	CADM1	11	115248355	C	T	0.46	0.89 (0.85–0.94)	7.33 × 10^−6^	0.48	0	4.66	NA	CADM1 (Lung)
rs1457538	PTPRO	12	15584196	G	C	0.26	0.86 (0.81–0.91)	9.68 × 10^−8^	0.36	8.49	1.96	7	PTPRO (Adipose tissue)
rs915378	MIR656	14	101713910	T	C	0.37	0.89 (0.84–0.94)	7.92 × 10^−6^	0.18	28.3	3.14	5	

APIP, apoptotic peptidase activating factor 1 interacting protein; AS3MT, arsenite methyltransferase; AUTS2, activator of transcription and developmental regulator AUTS2; BP, base position (hg19); CADD, Combined Annotation Dependent Depletion; CADM1, cell-adhesion molecule 1; CAST, calpastatin; CHR, chromosome; CI, confidence interval; CTNND2, catenin delta 2; eQTL, expression quantitative trait locus; ERAP1, endoplasmic reticulum aminopeptidase 1; GWAS, genome-wide association study; I2, heterogeneity; LINC00290, long intergenic non-protein-coding RNA 290; MAF, minor allele frequency; MIR656, microRNA 656; MKI67, marker of proliferation Ki-67; NA, not available; OR, odds ratio; PDHX, pyruvate dehydrogenase complex component X; PDZD2, PDZ domain containing 2; PTPRO, protein tyrosine phosphatase receptor type O; Q, Cochran’s Q statistics; RDB, RegulomeDB 2.0; SFXN2, sideroflexin 2; SNP, single-nucleotide polymorphism; WBP1L, WW domain binding protein 1-like.

**Table 2:** Age-standardized characteristics according to PRS in thirds among US males and females in the NHS1 and HPFS.

	PRS of NHS1			PRS of HPFS		
	Low (*n* = 3990)	Intermediate (*n* = 4022)	High (*n* = 4086)	Low (*n* = 2559)	Intermediate (*n* = 2463)	High (*n* = 2533)
Age (years)	57.2 (6.8)	56.9 (6.8)	57.2 (6.9)	57.6 (8.5)	57.3 (8.7)	57.8 (8.9)
Caucasian (%)	99.7	99.8	99.7	94.6	94.9	95.6
BMI (kg/m^2^)	23.6 (2.7)	23.7 (2.7)	23.6 (2.6)	24.9 (2.3)	24.8 (2.2)	24.9 (2.2)
Weight (kg)	63.7 (8.5)	63.8 (8.4)	63.6 (8.4)	79.5 (8.9)	79.3 (9.0)	79.5 (9.0)
Never smokers (%)	40.9	45.2	48.2	50.8	49.5	49.8
Past smokers (%)	39.5	37.1	35.4	42.2	42.4	42.3
Current smokers (%)	19.3	17.5	16.2	7.1	8.1	7.9
Alcohol intake (g/day)	8.4 (11.6)	7.3 (10.8)	6.6 (9.8)	12.2 (15.5)	12.8 (16.1)	11.9 (15.2)
Physical activity (MET-h/week)	15.7 (21.6)	15.2 (18.7)	14.7 (20.8)	21.8 (25.8)	20.6 (23.0)	20.4 (23.7)
Total energy intake (kcal/d)	1673.2 (466.1)	1754.4 (467.7)	1865.5 (487.4)	2002.4 (581.9)	2004.0 (594.7)	2058.0 (632.8)
AHEI	47.4 (10.2)	46.0 (9.9)	44.9 (9.7)	46.9 (10.9)	47.1 (11.1)	46.6 (10.7)
Glycemic load	96.9 (17.9)	99.0 (17.6)	100.1 (16.2)	124.0 (25.3)	123.9 (25.2)	124.5 (25.1)
Trans fat	1.8 (0.6)	1.9 (0.6)	2.0 (0.6)	2.8 (1.1)	2.8 (1.1)	2.9 (1.1)
Total fiber	17.6 (5.0)	17.4 (4.8)	17.1 (4.5)	21.2 (6.9)	21.2 (7)	20.7 (6.5)
Fruit	73.2 (44.8)	74.2 (44.3)	77.7 (45.5)	61.7 (147.1)	67.4 (160.2)	78.0 (187.1)
Vegetable	87.3 (49.5)	85.0 (45.5)	85.4 (44.2)	80.3 (214.2)	101.9 (280.9)	119.8 (315.1)
Coffee	17.0 (30.6)	17.4 (29.3)	18.3 (30.1)	10.3 (36.1)	9.9 (35.6)	12.2 (40.3)
Tea	6.5 (6.2)	6.1 (5.3)	6.3 (5.4)	7.3 (29.5)	9.7 (35.7)	11.5 (39.4)
Sweetened beverage	36.5 (39.8)	38.1 (42)	38.4 (40.7)	12.8 (24.9)	13.9 (28.8)	15.1 (31.9)
Chocolate	1.2 (3.5)	1.5 (3.8)	1.8 (4.3)	1.8 (5)	2.6 (10.8)	4.9 (18.1)
Ice cream	1.4 (2.6)	1.5 (2.6)	1.7 (2.8)	7.5 (29.9)	7.2 (29)	9.1 (33.2)
Cake	1.6 (2.1)	2.0 (3.2)	2.5 (3.4)	1.6 (15.3)	4.1 (27.6)	13.6 (53.3)
Sleep (h/day)	7.0 (0.9)	7.1 (0.9)	7.0 (0.9)	7.1 (0.8)	7.1 (0.8)	7.1 (0.8)
PRS	631.3 (8.0)	647.9 (3.8)	664.6 (8.1)	631.3 (7.5)	647.7 (3.8)	665.2 (9.0)
Cases/person years	729/76,338	749/78,784	693/80,321	330/47,722	331/46,575	312/47,416
Crude incidence/100 K PY	955	951	863	692	711	658

AHEI, alternate healthy eating index; BMI, body mass index; HPFS, Health Professionals Follow-up Study; MET, metabolic equivalent; NHS1, American Nurses’ Health Study; PRS, polygenic risk scores; PY, person-year. Value is not age-adjusted; data are presented as the mean (SD) for continuous variables, percentages for categorical variables, and are standardized to the age distribution of the study population.

**Table 3:** Adjusted HR (95% CI) of obesity for genotypes of the SNP rs4861982 in the NHS1 and HPFS ^1^.

	Model 1 (Age-Adjusted Model) ^2^			Model 2 (Multivariate-Adjusted Model) ^3^		
Independent Variable	HR	95% CI	*p*-Value	HR	95% CI	*p*-Value
rs4861982 (TG vs. GG)						
NHS1 ^4^	1.037	0.943–1.142	0.453	1.033	0.938–1.137	0.511
HPFS ^5^	1.010	0.873–1.168	0.897	0.992	0.858–1.148	0.914
Pooled ^6^	1.029	0.950–1.115	0.485	1.020	0.942–1.106	0.623
rs4861982 (TT vs. GG)						
NHS1 ^4^	1.103	0.857–1.419	0.448	1.111	0.863–1.430	0.414
HPFS ^5^	1.596	1.145–2.225	0.006	1.565	1.122–2.184	0.008
Pooled ^6^	1.262	1.032–1.543	0.023	1.259	1.030–1.540	0.025

^1^ CI, confidence interval; HPFS, Health Professionals Follow-up Study; HR, hazard ratio; NHS1, Nurses’ Health Study1; SNP, single-nucleotide polymorphism. ^2^ Model 1 is age-adjusted. ^3^ Model 2 is adjusted for age, smoking status, alcohol consumption, physical activity, trans fat, total energy intake, and race. ^4^ Follow-up in NHS1 was from 1986 to 2014. ^5^ Follow-up in HPFS was from 1986 to 2014. ^6^ Results of two cohorts are pooled by means of inverse variance-weighted, fixed-effects meta-analysis (all *P*-values for heterogeneity).

## Data Availability

The original contributions presented in the study are included in the article, further inquiries can be directed to the corresponding authors.
